# Workplace-based faculty development model: A meta-synthesis review of qualitative research

**DOI:** 10.12669/pjms.41.8.12470

**Published:** 2025-08

**Authors:** Rahila Yasmeen, Muhamad Saiful Bahri Yusoff

**Affiliations:** 1 Rahila Yasmeen, MHPE *First Affiliation:* Dean Riphah Academy of Research and Education, Riphah International University, Islamabad, Pakistan. *2nd Affiliation:* School of Medical Sciences, Universiti Sains Malaysia, Kelantan, Malaysia; 2 Muhamad Saiful Bahri Yusoff, PhD Faculty of Medicine and Health Sciences, University Putra Malaysia, Serdang, Selangor, Malaysia

**Keywords:** Workplace-Based Faculty Development, Learning Transfer, Peer Coaching, Cognitive Apprenticeship model, Meta-synthesis review

## Abstract

**Objective:**

To develop a model for workplace-based faculty development (WBFD) by identifying key components for its framework that enhance the transfer of teaching skills in the workplace through a meta-synthesis review of relevant qualitative studies in medical education.

**Methodology:**

We conducted a meta-synthesis using a meta-ethnographic approach. We searched PubMed, Medline, PsycINFO, SCOPUS and Google Scholar for qualitative studies. Studies published between January 2000 to September 2024 were included. We used a thematic synthesis approach to analyses the data, compare the findings from different studies and identified main themes for making the WBFD model.

**Results:**

From the 1,235 studies screened, 10 met the inclusion criteria for this review. Seven core themes emerged as key components of a framework for WBFD model: (1) Peer Coaching, (2) Learning by Observing, Doing and Reflecting, (3) Workplace-Based Learning, (4) Cognitive Apprenticeship Model, (5) Institutional Support, (6) Feedback and Reflection and (7) Evaluation of Faculty Development Programs. Peer coaching facilitated the faculty development through reflection and feedback, while the Cognitive Apprenticeship Model enhanced the learning transfer and provided scaffolding mechanisms that supported faculty in refining their teaching methodologies with institutional support.

**Conclusions:**

This meta-synthesis highlighted the significance of the WBFD model by integrating peer coaching with feedback, reflective practice, cognitive apprenticeship and institutional support with evaluation. The WBFD model promotes the sustainable learning transfer of teaching skills at the workplace. Future studies should examine how well this model works through experimental research on faculty development.

## INTRODUCTION

Faculty development is essential for improving teaching practices. Its importance becomes even more pronounced within workplace-based learning environments. In the past, faculty development has mostly used structured workshops and seminars. However, these conventional methods have often been found lacking in their capacity to effect sustained improvements in teaching practices.^1^,^2^ Key limitations include the absence of contextual learning, insufficient peer support and the lack of reinforcement mechanisms, which hinder the effective transfer of newly acquired teaching strategies.^3^,^4^

This challenge is particularly prominent in Pakistan, where studies observed that traditional faculty development programs often fail to yield long-term improvements in teaching effectiveness, especially in clinical settings.^5^,^6^ This highlights the need for more integrated, workplace-based faculty development (WBFD) strategies that can bridge the gap between theoretical knowledge and practical teaching competencies. WBFD helps faculty build their skills in real teaching situations while learning together with peers.^5^,^6^

Peer coaching is a key part of WBFD and supports faculty development. Studies highlight the positive impact of peer coaching and mentorship programs on faculty development.^7^ Through structured feedback and mutual support, peer coaching facilitates professional growth, fosters reflective practices and promotes the exchange of teaching strategies.^3^,^8^ Additionally, the role of reflective critique in facilitating learning transfer and behavioral change among faculty further validates the importance of ongoing reflection within WBFD.^5^

The need for workplace-integrated learning strategies is further supported by Lave’s Situated Learning Theory and Bandura’s Social Learning Theory, both of which suggest that learning is most effective when embedded within real-world contexts. This is often informal, as is the case with WBFD.^9^,^10^ According to Steinert et al., learning within the workplace not only enhances instructional competencies but also aligns teaching methods with the dynamic challenges faced in clinical and educational settings, integrating both formal and informal faculty development approaches.^1^

Furthermore, institutional support plays a critical role in the success of WBFD initiatives.^3^,^4^ Research by Garcia et al. and Rehman et al. found that institutions that actively support WBFD through mentorship programs and by reducing workload barriers report higher faculty satisfaction and improved teaching outcomes.^3^,^4^ Furthermore, Khan et al. identified that institutional policies that integrate faculty development into career advancement frameworks play a vital role in sustaining faculty engagement with these programs.^6^

Many approaches exist in the literature on workplace-based faculty development (WBFD), however, there is a lack of a clear framework that identifies the essential components for effective learning transfer. Therefore, this study aimed to develop this model by identifying the main components of WBFD that support teaching skills in faculty, by reviewing the existing literature.

## METHODOLOGY

We carried out a qualitative meta-synthesis that used a meta-ethnographic approach to analyses research on WBFD. We followed the PRISMA-ScR guidelines to conduct this review of qualitative studies. The study duration was six months, starting in September 2024 and ending in February 2025.

### Eligibility criteria:

We included studies with qualitative or mixed-methods designs, published between January 2000 to September 2024. We mainly included qualitative and mixed-methods studies but also selected mixed-methods research and systematic reviews that had enough qualitative data for this review. These studies gave useful insights for the meta-synthesis. Studies with only quantitative data were excluded.

### Ethical Considerations:

This study involved only previously published research. Therefore, formal ethical approval was not required. We followed ethical guidelines by being transparent in reporting and properly citing all sources.

### Search Strategy & Data Sources:

We searched PubMed, Medline, PsycINFO, SCOPUS and Google Scholar for relevant studies. The search included the following terms: ‘Faculty Development’, ’Workplace-Based Faculty Development’, ’Medical Education’, ’Learning Transfer’ and ’Peer Coaching’. Boolean operators (AND/OR) were used to refine the results and ensure comprehensive coverage.

### Study Selection & Screening:

Two reviewers screened the search results and any differences were settled by discussion. Full-text articles of potentially eligible studies were further reviewed to confirm their suitability for inclusion.

### Quality Assessment:

We followed the Framework for Assessing Qualitative Evaluations to check the quality of each study, focusing on research design, data collection and theme relevance.

### Data Extraction & Synthesis:

Thematic synthesis was used for data analysis. Three researchers independently coded the studies and later reviewed the themes together to ensure consistency. Only studies that met the quality benchmarks were included. We documented key details of each study in a summary table. It included the following details; author, year, study design, intervention type, outcomes, context and participants. A line-by-line coding process was performed to identify recurring themes across studies. The analysis followed a seven-step meta-ethnographic approach as outlined by Britten, Campbell, Pope, Donovan, Morgan and Pill^11^ and Noblit and Hare^12^ shown in Table-I.

**Table-I:** Seven-Step Process Used in the Meta-Synthesis Methodology.

S. no	Steps	Process
1	Getting Started	From the literature review, the researcher decided to address the question of “What are the ELEMENTS of workplace-based faculty development approaches?”
2	Deciding what is relevant to initial interest	Based on the question, we set appropriate search terms, criteria and data bases as shown below. The search terms were checked and refined by the corresponding author through library. Search terms: “faculty development’’ AND ‘’workplace-based learning’’, ‘’formal faculty development’’ AND ‘’formal faculty development’’, ‘’evaluation of faculty development’’ (‘’medical faculty’’ OR ‘’staff’’)
		Criteria:
		a.Peer-reviewed journal / article / book / thesis
		b.English Language
		c.Dated from 1980 – 2024
		d.Types of studies:
		i.Grounded Theory
		ii.Phenomenology
		iii.Ethnography
		iv.Action Research
		v.Case Study
		vi.Mixed Method Research
		e.Participants: Medical doctor (ranging from intern to consultant)
		Exclusion criteria:
		a.Commentary
		b.Qualitative or mixed method studies on intern shadowing / medical students
		c. d.Qualitative or mixed method studies on academicians
		Databases:
		a.Google Scholar (Citation indexes)
		b.PubMed, PubMed Central, National Library of Medicine (Subject data base)
		c. d.Medline (Subject data base)
		e.PsycINFO (Subject data base)
		f.SCOPUS (Citation indexes)
3	Reading the studies	The search results, reviewed by the researcher and one of the senior expert faculty members independently. It included all papers and the final agreement was achieved on inclusion by consensus. The researchers read the articles with the help of AI assistance and reach mutual consensus on the selected studies. They assess the quality by using the 18 items checklist from Framework for Assessing Qualitative Evaluations (Spencer, Ritchie, Lewis, & Dillon, 2003). The main themes were identified by the researcher from the selected primary studies.
4	Determining how the studies are related	The researcher then created a table mentioning the identified Author(s) and year, Invention type, Learning outcomes, Context and participants, key findings / themes. The researcher then examined line by line coding, generating themes across the selected studies.
5	Translating the studies into one another	In this step, the researcher systematically compared the identified themes and then examined the interpretation of the themes, range for reciprocal translation (similarities) and refutational translation (differences) in the contexts of the selected primary studies.
6	Synthesizing translations	The researcher in this step conducted the second level of synthesis where overarching themes were formed from Step 5. Then the researcher developed a line of argument to represent the common themes of ELEMENTS of WBFD along with its evaluation model. Themes were validated by the senior peer expert faculty of medical education
7	Expressing the synthesis	In the last step, the researcher communicated the line of argument into a more comprehensible format such as a framework for the stakeholders such as medical faculty, educationists and policy makers. The researcher used Confidence in the Evidence from Reviews of Qualitative Research (GRADE-CERQual) (Appendix B) for the assessment of confidence in the synthesis (Lewin et al., 2018).

We analyzed the included studies by comparing their findings, looking for both similarities and differences. To develop key themes, we applied reciprocal translation to highlight common findings, refutational translation to explore any conflicting results and line-of-argument synthesis to construct overarching themes from the data. Key study details, including author, year, intervention type, outcomes and main findings, were summarized in a structured table to support thematic analysis and comparison. This table facilitated cross-study comparisons and thematic synthesis (Table-I).

## RESULTS

### Search Results and Study Selection:

**Fig.1:**
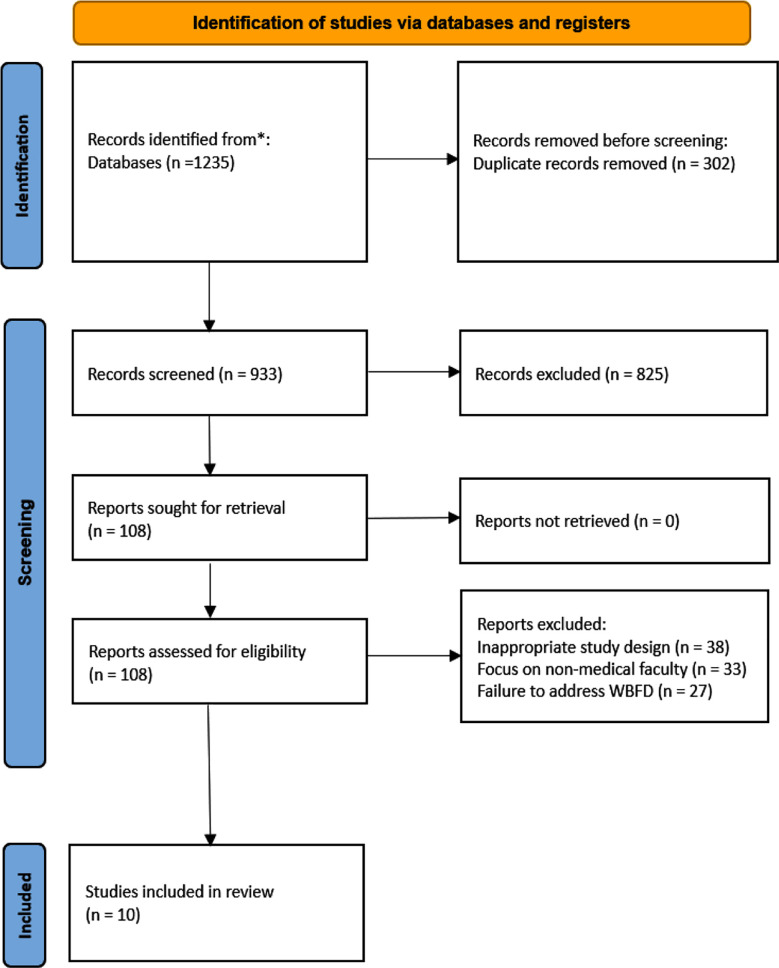
PRISMA Flowchart (A flowchart summarizing the study selection process is included here, following the PRISMA guidelines.

A systematic search from January 1, 2000 to December 31, 2024, found 1,235 studies in five major research databases. After a thorough review process, 10 studies were selected for inclusion in the final synthesis (Fig.1). Of the 1,235 studies found, 1,225 were excluded after screening titles and abstracts. They were excluded because they were not about faculty development, focused on undergraduate or non-medical faculty, used only quantitative methods, or did not cover workplace-based learning. These criteria were applied consistently by independent reviewers during the screening process.

### Characteristics of Included Studies:

The selected studies covered faculty development methods in different countries, including Australia, Canada, Saudi Arabia, the USA, Switzerland, Iran and Germany (Table-II). The studies had qualitative research, quantitative studies, systematic reviews and meta-analyses methods. The key findings from these studies highlight the importance of peer coaching, feedback mechanisms, learning through observation and practice and institutional support in improving faculty development and teaching effectiveness.^13^,^14^

**Table-II:** Characteristics of Included Studies.

Authors & Year	Country	Subgroups	Number of Participants	Type of Study	Key Findings	Qualitative Grading
Ladyshewsky & Sanderson (2021)^13^	Australia	Clinical educators	10	Qualitative	Best practices in peer coaching to enhance reflective practices and professional growth.	B
Sargeant et al. (2008)^14^	Canada	Medical educators	28	Qualitative	Peer feedback fosters reflective teaching.	B
Abdulghani et al. (2014)^20^	Saudi Arabia	Faculty across institutions	Systematic review	Systematic review	Demonstrated utility of multi-level training evaluation.	A
Jackson et al. (2018)^19^	USA	Multidisciplinary faculty	26	Mix method	Identified barriers and facilitators of learning transfer.	A
Steinert (2010)^16^	Canada	Novice and senior educators	32	Qualitative	Importance of real-world teaching contexts.	B
Garcia et al. (2017)^3^	Switzerland	PBL tutors	12	Mix method	Peer feedback enhances facilitation skills and reflective practices.	c
Kohan et al. (2023)^15^	Iran	Faculty in training	34	Systematic review	Highlights training relevance and alignment with goals as key factors for learning transfer.	A
Collins et al. (1991)^18^	USA	Medical trainees	N/A	Qualitative	Emphasizes mentorship, scaffolding and real-world teaching.	B
Burgess et al. (2020)^17^	Australia	Clinical educators	36	Qualitative	Highlights the importance of structured feedback and its role in clinical education to enhance reflective practices and teaching quality.	A
Tonhäuser & Büker (2016)^29^	Germany	Multidisciplinary faculty	Meta-analysis	Meta-analysis	Explores factors influencing skill transfer in workplace-based training programs.	A

### Themes Identified in the Meta-Synthesis:

The synthesis identified several key themes that are central to the WBFD model’s framework, shown in Table-III. These include peer coaching, feedback and reflection, learning through observation and practice, workplace-based learning, cognitive apprenticeship, institutional support, feedback and reflection and evaluation of faculty development programs. The themes provide insights into the components that contribute to effective WBFD.^15^,^16^

**Table-III:** Summary of themes derived from the meta-synthesis.

Original Themes (First Order)	Subthemes (Second Order)	Final Themes (Third Order)
Group discussion 1,2 Shared reflections 1 Reciprocal dialogue 2	Collaboration	Peer Coaching
Supporting junior faculty 1 Co-teaching experiences 1 Role-modelling positive feedback 3	Mutual Growth	
Constructive peer evaluations 2 Building confidence in teaching 2	Professional Development	
Watching experienced faculty teach 1 Students’ reaction to different teaching styles 4 Shadowing senior faculty 1	Observation	Learning by Observing, Doing and Reflecting
Simulation Exercises 3 Practising skills in a safe environment 5 Improve skills 6 case-based discussions 7	Hands-on learning	
Writing self-reflective 2 Identifying strengths and areas for improvement 5 Tracking growth 5	Reflection	
Teaching rounds in clinical settings 1 unpredictable learning environments 6 Managing complex patient cases with students 8	Real-World Challenges	Workplace-Based Learning
Teaching in fast-paced environments (e.g., emergency rooms) 6 on-the-job coaching 5 professional development^3^ Learning Environment for growth^3^	Embedded Learning	
Learning under expert supervision 7 Handling Challenges 8	Mentorship	Cognitive Apprenticeship Model
Breaking complex skills into manageable steps 7 Just-in-time guidance for trainees 5	Scaffolding	
Leadership buy-in for faculty development 8 Aligning goals with institutional mission 6	Leadership Advocacy	Institutional Support
funding 3 dedicated time for professional development 4	Institutional Resources	
structured feedback from peers^2^ Refining teaching approach 9 Modify teaching strategies 5 post-teaching debriefs 4 Reinforce key takeaways 1	Feedback Mechanisms	Feedback and Reflection
Self-assessing performance 6 dedicated time for professional development 4	Reflective Practices	
progress after training 8 confidence and skills 10 Improvements in teaching methods 9	Measuring Training Impact	Evaluation of Faculty Development Programs
clear goals 3 structured evaluations 8 performance over time 4	Defining Competency Standards	
applying training in real settings 9 shaping institutional learning culture 3 asting benefits of faculty programs 10	Long-Term Effectiveness	

Primary studies: 1^13^, 2 14, 3 15, 4 17, 5 3, 6 16, 7 18, 8 20, 9 19, 10^30^

### Theme-1: Peer Coaching:

Peer coaching is a significant formal method for faculty development. It supports teamwork, shared learning and professional growth. Faculty take part in feedback and reflection sessions with peers to improve their teaching. Peer coaching helps faculty to critically assess and improve their teaching in a supportive, non-hierarchical environment.^13^,^17^

### Theme-2: Learning by Observing, Doing and Reflecting:

Situated Learning Theory suggests that faculty learn best in real-world teaching settings. The studies show that faculty who watch skilled teachers, try new methods and reflect on their work are more likely to remember and use what they learn. This hands-on approach helps them develop effective teaching skills and adapt them to their specific teaching contexts.^14^,^18^

### Theme-3: Workplace-Based Learning:

Faculty development is most effective when it is integrated into daily work activities. Learning within the work environment allows faculty to deal with real challenges, which helps build confidence and competence in their teaching roles.^15^

### Theme-4: Cognitive Apprenticeship Model:

The Cognitive Apprenticeship Model emphasizes mentorship and scaffolding. In this model, new educators learn from experienced mentors, which helps them build teaching skills over time. It shows the value of learning by watching, practicing and reflecting to connect theory with practice.^18^

### Theme-5: Institutional Support:

Institutional support is important for faculty development. Successful programs need the institution’s commitment, including time, funding and staff. Studies indicate that faculty members are more likely to implement new teaching skills when they have institutional backing, including encouragement from supervisors and colleagues.^19^

### Theme-6: Feedback and Reflection:

Peer faculty feedback with reflections involves structured and informal processes where colleagues review each other’s teaching methods and practices to provide constructive suggestions for improvement at the workplace. This feedback mechanism promotes reflective teaching, critique and continuous professional growth, facilitating the transfer of teaching skills.^19^

### Theme-7: Evaluation of Faculty Development Programs:

Evaluating faculty development programs is important to assess their effectiveness. We found that assessing faculty development programs on various levels including faculty satisfaction, behavioral changes and institutional impact, offers a more comprehensive understanding of their effectiveness. Longitudinal assessments are also crucial to determine the long-term impact of these programs on teaching practices.^20^ Table-IV shows the summary of themes derived from the meta-synthesis.

**Table-IV:** Summary of themes derived from the meta-synthesis.

Themes Emerged	Methodological Limitations	Relevance	Coherence	Adequacy	CERQual Confidence
Peer Coaching	Minor concerns (variability in participant recruitment)	No concern	Minor concerns (consistent findings across studies)	Minor concerns (limited data depth)	High
Feedback & Reflection	Minor concerns (variability in methodological approaches)	Moderate concerns	Moderate concerns (some inconsistencies)	No concern	Moderate
Learning by Doing and Observing	No concern	No concern	Minor concerns	No concern	High
Workplace-Based Learning	Moderate concerns (limited robust studies on integration)	No concern	Moderate concerns	Minor concerns	Moderate
Cognitive Apprenticeship Model	Minor concerns (limited diversity of contexts)	No concern	Minor concerns	No concern	High
Institutional Support	Minor concerns (variability in methodological approaches)	Moderate concerns	Moderate concerns (some inconsistencies)	No concern	Moderate
Evaluation of Faculty Development Programs	High concerns (limited examples of long-term evaluations)	Moderate concerns	Moderate concerns	Minor concerns	Moderate

**Fig.2:**
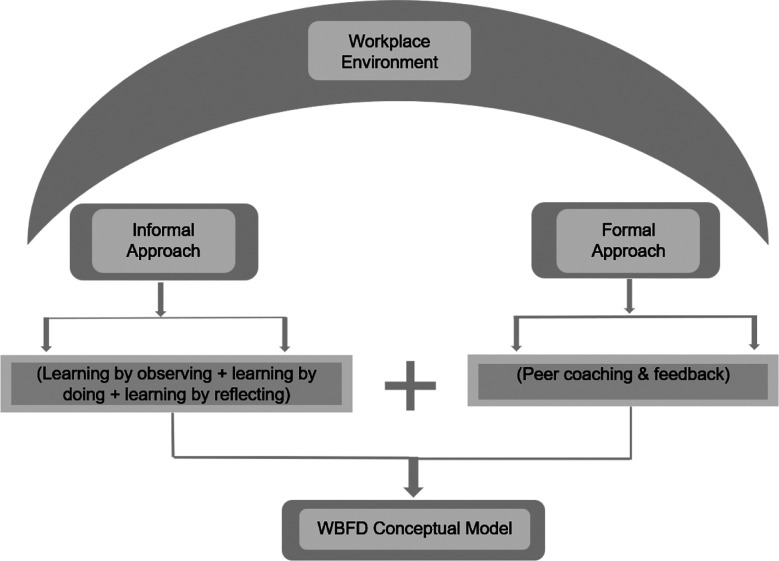
Workplace based faculty development Model framework.

The synthesis identified the key components for an effective WBFD model, integrating themes from the meta-analysis. Fig.2 illustrates the framework, highlighting the importance of institutional support and evaluation in planning and implementing WBFD programs.

## DISCUSSION

This meta-synthesis identified seven key components of the Workplace-Based Faculty Development (WBFD) model: peer coaching, learning through observation, practice and reflection, workplace-based learning, the cognitive apprenticeship model, institutional support, feedback and reflection and systematic evaluation of faculty development programs. Together, these components create a model that supports learning within the faculty’s everyday work environment. By combining workplace-based learning with peer support, feedback and institutional backing, the WBFD model encourages the practical use and ongoing improvement of teaching skills in real settings.

Peer coaching plays a key role in faculty development by providing continuous support and feedback, which encourages reflection and helps faculty enhance their teaching.^4^,^13^,^21^,^22^ In the context of WBFD, peer coaching provides faculty with avenues to collaborate and learn from each other in real-time teaching scenarios.^17^,^23^ Rehman et al. demonstrated that mentorship and peer coaching programs at an institution were effective in improving faculty engagement and satisfaction, leading to improved teaching outcomes.^24^ Moreover, the positive impact of peer coaching has been emphasized by Wajid et al., where Cognitive Peer Coaching (CPC) was shown to enhance facilitation skills and promote reflective thinking among faculty.^7^ In this context, the cognitive apprenticeship model supports peer coaching by offering a structured process of demonstration, guidance, support and gradual transfer of responsibility. This approach guides faculty from observing to teaching on their own, with continued support from experienced colleagues.

The findings also show that learning by observing and practicing in real teaching settings is important. Faculty development programs that prioritize hands-on practice enable educators to observe experienced mentors, experiment with teaching strategies and engage in reflective practice. This helps them understand teaching better and improve over time. Embedding learning within the work environment, rather than relying solely on traditional classroom-based training, leads to better retention and application of new knowledge.^14^ This focus on real-world practice reflects Lave’s Situated Learning Theory, which explains that learning is most effective when it occurs in the same setting where it will be used. In workplace-based faculty development, observation and peer interaction play a key role, as described in Bandura’s Social Learning Theory, which emphasizes learning through observing others and adopting their behaviors.

Feedback and reflection are fundamental for continuous improvement in teaching. Faculty members who engage in structured feedback sessions and self-reflection exercises tend to make more significant improvements in their teaching skills.^4^ Kiran et al. emphasized that immediate and structured feedback plays a pivotal role in translating knowledge into practice, particularly when faculty receive feedback that is specific and actionable.^8^ Additionally, Amin et al. noted that using reflective practices, like keeping a teaching portfolio or journal, helps educators track their progress, find areas to improve and strengthen their teaching. Rehman et al. further highlighted that incorporating reflection into faculty development programs can build greater confidence in instructional abilities, leading to more effective teaching.^24^ In the WBFD approach, structured feedback and reflection are supported by the cognitive apprenticeship model, where feedback is built into the ongoing mentoring and skill development process.

Institutional support is key for the success of workplace-based faculty development (WBFD) programs. These programs are more effective when institutions prioritize teaching, allocate time and funding and foster a culture of continuous learning.^4^,^19^,^25^ Khan et al. found that linking faculty development to career growth helps improve teaching in the long run.^26^ However, barriers like heavy workloads and lack of incentives can make it harder for faculty to apply new skills. In contrast, institutions that offer mentoring programs and reduce workload barriers create an environment where faculty members feel supported and motivated to improve their teaching.^7^,^24^ To overcome these barriers, institutions should provide flexible scheduling for development activities, formally recognize faculty participation and link development efforts to faculty appraisal and promotion. These strategies may support sustained faculty engagement and help reduce resistance to participation.

Evaluating faculty development programs is important to see how effective they are. Traditional models, such as the Kirkpatrick Model, tend to focus mainly on short-term learning outcomes. Newer approaches recommend using both qualitative and quantitative data for a fuller evaluation.^20^,^27^,^28^ Kiran et al. recommended shifting the focus from immediate feedback to evaluating the long-term impact of faculty development.^8^ This requires multi-level assessments that consider satisfaction, learning outcomes, behavioral changes and institutional impact. Amin et al. emphasized the value of longitudinal evaluations, which track the long-term effects of faculty development programs on teaching practices, helping institutions refine and improve their development strategies.^5^ In the WBFD model, evaluation is seen as an ongoing part of faculty development, rather than a separate activity. Institutions can track faculty progress by monitoring their teaching performance over time. They can also identify areas that need improvement based on observations and feedback. Development efforts may be adjusted by using regular feedback, structured reflection and assessments at different levels. The primary aim was to enhance teaching practices and institutional policies rather than only focusing on participant satisfaction.

### Limitations:

This review included only ten studies and therefore limits the extent to which the findings can be generalized across different contexts. Further research is needed to see whether the WBFD model is applicable in different educational settings. Moreover, it was difficult to assess sustained outcomes as many of the included studies did not have a long term follow up. It is important to note the possibility of publication bias, as studies with negative or inconclusive results may not have been published.

Moreover, different contextual settings, such as different cultures across institutions and institutions from different countries with varied economic backgrounds might also affect how WBFD program’s function. Future research should further investigate these factors. The shift towards online and hybrid education after the pandemic has highlighted the need to adapt faculty development activities for virtual platforms. Essential elements of the WBFD model; peer coaching, feedback and reflection, can be implemented through online mentoring, virtual discussions and remote observation. These strategies may offer a practical solution for institutions with limited access to on-site training, helping them sustain faculty development despite resource constraints.

## CONCLUSION

This review highlights key components that contribute to effective workplace-based faculty development (WBFD) programs. Five main components were identified for improving development of faculty and teaching practices; peer coaching, hands-on learning, structured feedback, reflection and institutional support. We found that a culture of collaboration and continuous professional growth can be encouraged by incorporating the social learning theories and the cognitive apprenticeship model. We can improve teaching quality and even sustain faculty engagement and institutional development by strengthening WBFD initiatives via structured mentorship, workplace-based learning opportunities and systematic evaluations.
